# Interaction between plasma fetuin‐A and free fatty acids predicts changes in insulin sensitivity in response to long‐term exercise

**DOI:** 10.14814/phy2.13183

**Published:** 2017-03-07

**Authors:** Sindre Lee, Frode Norheim, Hanne L. Gulseth, Torgrim M. Langleite, Kristoffer J. Kolnes, Daniel S. Tangen, Hans K. Stadheim, Gregor D. Gilfillan, Torgeir Holen, Kåre I. Birkeland, Jørgen Jensen, Christian A. Drevon

**Affiliations:** ^1^ Department of NutritionInstitute of Basic Medical SciencesFaculty of MedicineUniversity of OsloOsloNorway; ^2^ Division of CardiologyDepartment of MedicineUniversity of California at Los AngelesLos AngelesCalifornia; ^3^ Institute of Clinical MedicineFaculty of MedicineUniversity of OsloOsloNorway; ^4^ Department of Physical PerformanceNorwegian School of Sport SciencesOsloNorway; ^5^ Department of Medical GeneticsOslo University Hospital and University of OsloOsloNorway; ^6^ Department of EndocrinologyMorbid Obesity and Preventive MedicineOslo University HospitalOsloNorway

**Keywords:** Adipose tissue, exercise, fetuin‐A, free fatty acids, hepatokine, human, insulin sensitivity

## Abstract

The hepatokine fetuin‐A can together with free fatty acids (FFAs) enhance adipose tissue (AT) inflammation and insulin resistance via toll‐like receptor 4 (TLR4). Although some of the health benefits of exercise can be explained by altered release of myokines from the skeletal muscle, it is not well documented if some of the beneficial effects of exercise can be explained by altered secretion of hepatokines. The aim of this study was to examine the effect of interaction between fetuin‐A and FFAs on insulin sensitivity after physical exercise. In this study, 26 sedentary men who underwent 12 weeks of combined endurance and strength exercise were included. Insulin sensitivity was measured using euglycemic‐hyperinsulinemic clamp, and AT insulin resistance was indicated by the product of fasting plasma concentration of FFAs and insulin. Blood samples and biopsies from skeletal muscle and subcutaneous AT were collected. Several phenotypic markers were measured, and mRNA sequencing was performed on the biopsies. AT macrophages were analyzed based on mRNA markers. The intervention improved hepatic parameters, reduced plasma fetuin‐A concentration (~11%, *P < *0.01), slightly changed FFAs concentration, and improved glucose infusion rate (GIR) (~33%, *P < *0.01) across all participants. The change in circulating fetuin‐A and FFAs interacted to predict some of the change in GIR (*β *= −42.16, *P *=* *0.030), AT insulin resistance (*β *= 0.579, *P *=* *0.003), gene expression related to TLR‐signaling in AT and AT macrophage mRNA (*β *= 94.10, *P *=* *0.034) after exercise. We observed no interaction effects between FFAs concentrations and leptin and adiponectin on insulin sensitivity, or any interaction effects between Fetuin‐A and FFAs concentrations on skeletal muscle TLR‐signaling. The relationship between FFAs levels and insulin sensitivity seemed to be specific for fetuin‐A and the AT. Some of the beneficial effects of exercise on insulin sensitivity may be explained by changes in circulating fetuin‐A and FFAs, promoting less TLR4 signaling in AT perhaps by modulating AT macrophages.

## Introduction

The prevalence of obesity is increasing worldwide, representing an important public health problem (Finucane et al. 2011). Obesity increases morbidity and mortality, largely attributed to increased incidence of type 2 diabetes mellitus (T2DM) and cardiovascular disease (CVD) (Zimmet et al. 2001; Li et al. 2006), which have insulin resistance as a common and important risk factor (Saltiel and Kahn 2001; Olefsky and Glass 2010; Nolan et al. 2011; Fu et al. 2012).

Physical exercise substantially increases insulin sensitivity and represents one of the most important options to prevent and treat obesity. In the last decade, much attention has been paid to myokines produced and released from the skeletal muscle, which may play a role to increase in insulin sensitivity during and after physical exercise (Pedersen et al. 2007; Pedersen and Febbraio 2012). Moreover, an altered pattern of adipokine secretion from expanded and inflamed adipose tissue may be important for the regulation of insulin sensitivity in different tissues (Ouchi et al. 2011; Turer and Scherer 2012). Furthermore, physical exercise seems to reduce adipose tissue inflammation and reverse the dysregulation of several adipokines (Lee et al. 2016). In addition to muscle and fat tissue, the liver plays an important role in regulation of whole body insulin sensitivity. Several hepatokines may affect glucose and lipid metabolism and thereby the risk of developing T2DM and CVD (Stefan and Haring 2013b; Jung et al. 2016). However, it remains largely unknown if the beneficial effects of physical exercise can be explained by altered secretion of hepatokines.

Recently, much attention has been focused on fetuin‐A, which might be one of the most important hepatokines regulating human metabolism (Pal et al. 2012; Stefan and Haring 2013a,b). Fetuin‐A is a secretory glycoprotein produced in the liver, and it is a natural inhibitor of the insulin receptor tyrosine kinase in the liver as well as in the skeletal muscle (Auberger et al. 1989). Fetuin‐A serves as an adaptor protein for saturated fatty acid‐mediated activation of Toll‐like receptor 4 (TLR4) (Pal et al. 2012). The fetuin‐A‐fatty acid complex is particularly interesting because it might be a potential driving force behind T2DM and CVD by inducing inflammatory signaling and insulin resistance in adipose tissue (Pal et al. 2012). Fetuin‐A might also induce insulin resistance by itself, as well as together with FFAs, at least according to studies on overfeeding in humans (Samocha‐Bonet et al. 2014). Obese human participants with T2DM have increased levels of fetuin‐A in blood and increased adipose tissue expression of proinflammatory cytokines (Pal et al. 2012). Interestingly, free fatty acids (FFAs) may enhance increase proinflammatory cytokine expression and insulin resistance only in the presence of fetuin‐A in human adipocytes in vitro (Pal et al. 2012). Furthermore, this interaction is supported by human in vivo studies (Stefan and Haring 2013a). It has been proposed that fetuin‐A is the missing link in lipid‐induced inflammation (Heinrichsdorff and Olefsky 2012).

In our present study, we examine the effect of an interaction calculated statistically between plasma concentration of FFAs and fetuin‐A on insulin sensitivity after 12 weeks of physical exercise intervention (Langleite et al. 2016).

## Materials and Methods

Extensive details regarding the MyoGlu study are published elsewhere (Langleite et al. 2016).

### Ethical approval

The study adhered to the Declaration of Helsinki and was approved by the National Regional Committee for Medical and Health Research Ethics North, Tromsø, Oslo, Norway. The study was registered with the US National Library of Medicine Clinical Trials registry (NCT01803568). Written informed consent was obtained from all participants prior to any study‐related procedure.

### Participants and exercise intervention

Twenty‐six sedentary men were originally included in the study. Thirteen were classified as normoglycemic, normal weight controls and another 13 as dysglycemic, overweight. The criteria for these classifications were as follows: F‐glucose <5.6 mmol/L and 2‐h glucose <7.8 mmol/L and BMI 19–25 or F‐glucose ≥5.6 mmol/L and/or 2‐h glucose ≥7.8 mmol/L and BMI 27–32 (Langleite et al. 2016).

Four participants differed slightly from these predefined criteria, but the conclusions were similar irrespective of using 26 or 22 participants. The *P‐*value for the interaction effect between plasma fetuin‐A and FFAs levels with GIR increased with fewer data points, but the trend remained the same (*P *<* *0.1).

Exclusion criteria in this study were known hypertension, liver or kidney disease, chronic inflammatory disease, or any medication expected to affect glucose metabolism (lipid lowering, antihypertensive, ASA, corticosteroids, etc.).

The participants underwent combined resistance and endurance exercise for 12 weeks, including two endurance bicycle sessions (60 min) and two whole body resistance‐training sessions (60 min) per week. The exercise was strictly supervised and the participants were informed not to change their diet. The diet was monitored using food frequency questionnaires before as well as after the exercise intervention (Langleite et al. 2016).

### Insulin sensitivity

The euglycemic‐hyperinsulinemic clamp was performed after an overnight fast, a fixed dose of insulin 40 mU/m^2^ min^−1^ was infused, and glucose 200 mg/mL was adjusted to maintain plasma glucose levels at 5.0 mmol/L for 150 min (euglycemia) (DeFronzo et al. 1979). Insulin sensitivity is reported as glucose infusion rate relative to body weight (mg/kg/min) and relative to fat free mass (mg/FFM/min) during the last 30 min of the clamp. Full blood glucose concentration was measured by a glucose oxidase method (YSI 2300, Yellow Springs, OH), and plasma glucose concentration was calculated (full blood glucose × 1.119). The products of fasting plasma concentration of insulin and FFAs levels were calculated as an indicator of adipose tissue insulin resistance.

The participants refrained from physical exercise and alcohol for 2 or 3 days before pretests and posttests, respectively. Thus, the training sessions, clamp tests and biopsy samplings did not interfere with each other, and were performed under similar conditions at different days before as well as after the intervention.

### Blood and tissue sampling

Blood (*n *=* *26), muscle (*n *=* *26) and subcutaneous adipose (*n *=* *24, two participants in the dysglycemic, overweight group did not donate adipose tissue biopsies) tissue samples were taken before as well as after 12 weeks intervention (Langleite et al. 2016). Muscle biopsies were taken from m. vastus lateralis, subcutaneous adipose tissue biopsies were taken from the periumbilical area, and blood sampling was performed by standard antecubital venous puncture. Serum and EDTA‐added plasma samples were stored at −80°C until analyzed.

Biopsies from m. vastus lateralis were immediately transferred to RNA‐later (Qiagen, Limburg, Netherland), kept overnight at 4°C, and transferred to −80°C. Subcutaneous adipose biopsies were immediately frozen in liquid nitrogen and stored at −80°C until further processing.

Plasma levels of fetuin‐A (Catalog # DFTA00, R&D, MN) were measured in duplicates using enzyme‐linked immunosorbent assays (ELISA) according to the manufacture's protocols. Optical density was determined using a microplate reader (Titertec Multiscan Plus; EFLAB, Helsinki, Finland), set to 450 or 490 nm depending on the actual protocols. Standard curves were generated using best‐fit curves.

Free fatty acid levels were determined using a MAXMAT PL multianalyzer (MAXMAT SA, Montpellier, France) with relevant reagents (ref # D07940/D07950, DIALAB, Wiener Neudorf, Austria).

### Tissue RNA isolation and cDNA synthesis

Frozen biopsies were cooled in liquid nitrogen and crushed by a pestle to powder in a liquid nitrogen‐cooled mortar. Tissue powder was then transferred into 1 mL QIAzol Lysis Reagent (Qiagen), and homogenized using TissueRuptor (Qiagen) at full speed for 15 sec, twice. Total RNA was isolated from the homogenate using a miRNeasy Mini Kit (Qiagen). RNA integrity and concentration were determined using Agilent RNA 6000 Nano Chips on a Bioanalyzer 2100 (Agilent Technologies Inc, Santa Clara, CA). Using a high‐capacity cDNA Reverse Transcription Kit (Applied Biosystems, Foster City CA), 200 ng of total RNA was converted to cDNA for TaqMan real‐time RT‐PCR.

### RNA sequencing

Indexed sequencing libraries were prepared from RNA from all muscle and adipose tissue samples and deep‐sequenced using the Illumina HiSeq 2000 system (Illumina, San Diego, CA). Illumina HiSeq RTA (real‐time analysis) v1.17.21.3 was used for real‐time analysis during the sequencing. Reads passing Illumina's recommended parameters were demultiplexed using CASAVA v1.8.2. For prealignment quality checks, we used the software FastQC v0.10.1. The mean library size was ~44 millions unstranded single‐ended reads for muscle tissue and ~55 millions for adipose tissue with no difference between groups or time points. No batch effects were observed. cDNA sequenced reads alignment was done using Tophat v2.0.8, Samtools v0.1.18, and Bowtie v2.1.0 with default settings against the UCSC hg19 annotated transcriptome and genome dated 14 May 2013. Postalignment quality controls were performed using the Integrative Genome Viewer v2.3 and BEDtools v2.19.1. Reads counted by gene feature were performed by featureCounts in Rsubread 1.14.2 and presented as reads per kilobase of transcript per million mapped reads (RPKM).

### Liver fat measurements using magnetic resonance spectroscopy

Lipid and water content in the liver were sampled using three Single Voxel Proton Spectroscopy acquisitions. The lipid fractions were presented as arbitrary units (AU) based on measured amplitudes of lipid and water signals. The jMRUI software package (http://www.jmrui.eu) was used to analyze single voxel spectroscopy data. Time‐domain quantification of 1H short echo time signals was used for peak fitting. The water peak, sampled in a water phantom with the applied PRESS sequence, and the lipid peak, created a two‐peak basis set. This was modeled as a single peak using the jMRUI simulation tool (http://www.jmrui.eu). Soft constraints on frequency and dampening was used to fit the peaks. The lipid fraction (*f*) was calculated from the formula; *f *= *Al*(*Al *+ *Aw*)*,* where *Al* and *Aw* denotes the fitted amplitude of the lipid and water signals, respectively.

### Statistical analyses

Baseline characteristics and the effect of exercise were modeled using linear regression (Table 1). The included terms were the parameter tested as dependent variable and participants, due to repeated measures, and time point (baseline and 12 weeks) as independent variables. The formulas used were: parameter ~ group for group differences and parameter ~ participants + time point for exercise effects, corrected for repeated measures. The tilde (~) and plus (+) are Wilkinson‐Rogers symbolic description of factorial models for analysis of variance (ANOVA). To compare whether the changes in response to exercise differed between the groups, we compared delta values (12 weeks minus baseline values). These models also included baseline values as a covariate to correct for differences at baseline (Table 1).

**Table 1:** Participant characteristics at baseline and responses to exercise^a^

	Control		Dysglycemics	
	Baseline	Δ	Baseline	Δ
*n* (men)	13		13	
Age (y)	49.8 ± 2.1		52.5 ± 1.6	
HbA1c (%)	5.2 ± 0.1		5.5 ± 0.1^c^	
Systolic BP (mmHg)	128.7 ± 3.3		132.3 ± 2.4	
Diastolic BP (mmHg)	73.5 ± 2.3		81.9 ± 2.7^c^	
BMI (kg/h^2^)	23.5 ± 0.5	0.0 ± 0.1	29.0 ± 0.7^c^	−0.4 ± 0.3
Weight (kg)	78.5 ± 2.3	−0.3 ± 0.4	95.4 ± 2.8^c^	−1.7 ± 0.6^d^
fP‐insulin (pmol/L)	38.5 ± 5.2	0.2 ± 5.6	65.3 ± 7.5^c^	11.7 ± 7.8^e^
fP‐glucose (mmol/L)	5.0 ± 0.1	0.2 ± 0.1^d^	5.8 ± 0.1^c^	0.1 ± 0.1
GIR (mg/kg/min)	7.6 ± 0.4	2.7 ± 0.6^d^	4.2 ± 0.5^c^	1.2 ± 0.3^d^
GIR (mg/FFM/min)	9.4 ± 0.5	3.4 ± 0.7^d^	5.7 ± 0.7^c^	1.6 ± 0.4^d^
GIR (mmol/m^2^/min)	1659.2 ± 94.5	601.9 ± 123.7^d^	1004.2 ± 113.0^c^	283.3 ± 76.9^d^
Clamp‐insulin (pmol/L)	443.9 ± 36.6	6.6 ± 23.6	455.5 ± 20.2	56.3 ± 24.5^d^
fP‐ALAT (U/L)	24.7 ± 2.2	−1.1 ± 1.4	44.8 ± 6.8^c^	−11.7 ± 5.0^d^
fP‐ASAT (U/L)	18.2 ± 1.8	0.3 ± 1.0	24.2 ± 4.2	−6.2 ± 2.7^d^^,^ e
Liver fat (AU)^b^	2.8 ± 0.6	−2.8 ± 0.6^d^	9.1 ± 1.6^c^	−2.7 ± 0.7^d^^,^ e
fP‐hsCRP (mg/L)	1.0 ± 0.2	0.3 ± 0.3	2.8 ± 0.9	−0.8 ± 0.7
fP‐Fetuin‐A (ng/L)	445.8 ± 31.4	−54.1 ± 20.0^d^	491.6 ± 40.2	−44.5 ± 11.1^d^
fP‐FFA (mmol/L)	25.5 ± 4.0	−8.6 ± 3.9^d^	22.3 ± 2.2	0.5 ± 2.9^e^

Control; normal weight, normoglycemic men. Dysglycemics; overweight, dysglycemic men. fP, fasting blood plasma; FFA, free fatty acids; BP, blood pressure; ALAT, alanine transaminase; ASAT, aspartate transaminase; hsCRP, high‐sensitivity C‐reactive protein; GIR, glucose infusion rate. Open cells indicates values not available postintervention. Clamp‐insulin was measured as the average of three measurements during steady‐state of the clamp test.

a All comparisons were made using linear regression.

b *n *=* *10 dysglycemic men.

c *P *≤* *0.05 compared to control.

d *P *≤* *0.05 compared to baseline.

e *P *≤* *0.05 compared to the change in the control group, corrected for differences at baseline.

The second linear regression models were constructed to test the effect of changes in circulating plasma concentrations of fetuin‐A and FFAs, and their interaction, on changes of insulin sensitivity (Table 2). The included terms were insulin sensitivity as dependent variable and participants, group allocation, plasma fetuin‐A and FFAs concentration levels as independent variables. The group variable was included to correct for several differences between the groups (Table 1), instead of correcting all differing variables separately, to maximize the degrees of freedom in the models. The formula used to investigate the separate effect of plasma concentration of fetuin‐A or FFAs on insulin sensitivity was insulin sensitivity ~ participant + group + fetuin‐A (or FFAs), which predicted changes in insulin sensitivity as a function of changes in plasma fetuin‐A levels (or plasma FFAs levels). To test for interaction effects between changes in plasma concentrations of fetuin‐A and FFAs on changes in insulin sensitivity, we used the formula: insulin sensitivity ~ participant + group + fetuin‐A*FFAs. The latter variable (interaction between fetuin‐A and FFAs) was the term of interest, representing the fetuin‐A‐FFAs complex (Pal et al. 2012; Stefan and Haring 2013a). The same models were subsequently rerun substituting plasma concentrations of fetuin‐A levels with plasma concentrations of total adiponectin, high molecular weight adiponectin or leptin, to test if the interaction effect between fetuin‐A and FFAs were unique.

**Table 2:** Relationships between plasma concentrations of fetuin‐A and FFAs, and their interaction, with insulin sensitivity

	GIR (mg/kg/min)		GIR (mg/FFM/min)		GIR (mmol/m^2^/min)	
	*β *± SE^a^	*P*	*β *± SE	*P*	*β *± SE	*P*
Fetuin‐A						
(Intercept)	3.166 ± 0.539	0.000	3.894 ± 0.683	0.000	694.564 ± 122.712	0.000
Group^b^	−1.598 ± 0.632	0.019	−1.819 ± 0.800	0.033	−335.077 ± 143.840	0.029
Fetuin‐A	0.008 ± 0.006	0.177	0.010 ± 0.007	0.173	1.713 ± 1.279	0.194
FFAs						
(Intercept)	2.773 ± 0.521	0.000	3.384 ± 0.661	0.000	608.882 ± 118.309	0.000
Group	−1.554 ± 0.702	0.037	−1.756 ± 0.890	0.061	−325.948 ± 159.317	0.052
FFAs	0.348 ± 2.769	0.901	0.364 ± 3.512	0.918	81.271 ± 628.860	0.898
Fetuin‐A*FFAs						
(Intercept)	3.348 ± 0.562	0.000	4.118 ± 0.711	0.000	736.183 ± 127.472	0.000
Group	−1.937 ± 0.645	0.007	−2.244 ± 0.817	0.012	−413.011 ± 146.392	0.010
Fetuin‐A	0.006 ± 0.005	0.311	0.007 ± 0.007	0.306	1.196 ± 1.221	0.338
FFAs	−3.953 ± 3.230	0.234	−5.113 ± 4.088	0.225	−917.417 ± 732.889	0.224
Fetuin‐ A:FFAs	−0.182 ± 0.080	0.032	−0.232 ± 0.101	0.031	−42.160 ± 18.073	0.030

GIR; glucose infusion rate.

a SE; standard error.

b Dysglycemic, overweight men.

The third regression models were constructed to test the effect of changes in plasma concentrations of fetuin‐A and FFAs, and their interaction, on changes in TLR‐ or insulin‐signaling in adipose tissue and skeletal muscle (Tables 3, 4). The formulas used were either gene ~ participants + group + fetuin‐A (or FFAs) for simple effects, or gene ~ participants + group + fetuin‐A *FFAs for interaction effects. “Gene” represents a gene annotated in the Kyoto Encyclopedia of Genes and Genomes (KEGG), Biocarta or Reactome TLR‐ and insulin‐signaling pathways (http://software.broadinstitute.org/gsea/msigdb/). The number of significantly correlated genes with a parameter was compared to the total number of genes in the pathway using a hypergeometric distribution, whereby we tested if the number of overlapping genes were significantly larger than expected by chance. More details are presented in the legend to Tables 3, 4.

**Table 3:** TLR‐signaling pathways in adipose tissue may be related to plasma concentration of fetuin‐A and FFAs, and their interaction^a^

	K	k	k/K	*n*	*P‐*value
Fetuin‐A					
TLR‐signaling	118	20	0.17	1663	<0.001
Insulin receptor signaling	108	6	0.06	1663	0.777
FFAs					
TLR‐signaling	118	3	0.03	851	0.800
Insulin receptor signaling	108	4	0.04	851	0.546
Fetuin‐A*FFAs					
TLR‐signaling	118	10	0.09	1089	0.045
Insulin receptor signaling	108	5	0.05	1089	0.557

The *P‐*values were calculated from a hypergeometric distribution (k‐1, K, N‐K, n) where K, genes in gene set; k, genes in overlap; N, total number of genes tested (23.710); and n, total number of genes predicted by the model. The actual genes present in the overlaps (k) are presented in Figure 2.

a Reactome, Biocarta and KEGG pathways for toll‐like receptor (TLR) and insulin receptor signaling were merged into one gene set for TLR and insulin receptor signaling, respectively.

**Table 4:** Insulin receptor signaling in skeletal muscle may be related to plasma concentration of fetuin‐A levels^a^

	K	k	k/K	*n*	*P‐*value
Fetuin‐A					
TLR‐signaling	118	20	0.09	1430	0.099
Insulin receptor signaling	108	15	0.14	1430	0.002
FFAs					
TLR‐signaling	118	2	0.02	604	0.806
Insulin receptor signaling	108	1	0.01	604	0.939
Fetuin‐A*FFAs					
TLR‐signaling	118	7	0.06	1291	0.465
Insulin receptor signaling	108	4	0.04	1291	0.846

The *P‐*values were calculated from a hypergeometric distribution (k‐1, K, N‐K, n) where K, genes in gene set; k, genes in overlap; *N*, total number of genes tested (23.710); and n: total number of genes predicted by the model. The actual genes present in the overlaps (k) are shown in Figure 3.

a Reactome, Biocarta, and Kegg pathways for TLR and insulin receptor signaling were merged into one gene set for TLR and insulin receptor signaling, respectively.

The final regression models (Table 6) were constructed to test the effect of changes in circulating fetuin‐A and FFAs levels, and their interaction, on changes in gene markers of adipose tissue macrophages. The formulas used were marker ~ participants + group + fetuin‐A (or FFAs) for simple effects, and marker ~ participants + group + fetuin‐A *FFAs for interaction effects. “Marker” denotes the mean expression of several validated macrophage markers; macrophages in general, the M1‐like and M2‐like phenotype. The list of macrophage‐specific markers is presented in Table 5 and were obtained from previous studies of human adipose tissue (Capel et al. 2009; Ahlin et al. 2013) or based on the frequency of use in the literature (Hill et al. 2014). We previously evaluated the specificity of these markers, both the individual expressions and the mean expressions, by running comparisons in several white blood cell types (Lee et al. 2016).

**Table 5:** mRNA markers of macrophages in human adipose tissue based on previously published data (Capel et al. 2009; Ahlin et al. 2013; Hill et al. 2014)

Markers	Symbol	Description	Study
Ma^a^	ACP5	Acid phosphatase 5, tartrate resistant	(Capel et al. 2009; Ahlin et al. 2013)
	CCL22	C‐C motif chemokine ligand 22	
	CD68	CD68 molecule	
	CD163	CD163 molecule	
	CHIT1	Chitinase 1	
	CRABP2	Cellular retinoic acid‐binding protein 2	
	CSF1R	Colony‐stimulating factor 1 receptor	
	GLA	Galactosidase alpha	
	GM2A	GM2 ganglioside activator	
	IL1RN	Interleukin 1 receptor antagonist	
	LILRB4	Leukocyte immunoglobulin like receptor B4	
	LIPA	Lipase A, lysosomal acid type	
	MRC1	Mannose receptor, C type 1	
	MSR1	Macrophage scavenger receptor 1	
	PLA2G7	Phospholipase A2 group VII	
	PLA2G15	Phospholipase A2 group XV	
	SIGLEC1	Sialic acid‐binding Ig‐like lectin 1	
	SLC38A6	Solute carrier family 38 member 6	
M1‐like	CCL2	C‐C motif chemokine ligand 2	(Hill et al. 2014)
	TNF	Tumor necrosis factor	
	IL8	Interleukin 8	
	COX20	COX20 cytochrome c oxidase assembly factor	
	IL6	Interleukin 6	
	IL1B	Interleukin 1 beta	
	ITGAX	Integrin subunit alpha X	
	TLR4	Toll‐like receptor 4	
	CCR2	C‐C motif chemokine receptor 2	
	IL1RN	Interleukin 1 receptor antagonist	
M2‐like	IL10	Interleukin 10	(Hill et al. 2014)
	MRC1	Mannose receptor, C type 1	
	TGFB1	Transforming growth factor beta 1	
	CCL18	C‐C motif chemokine ligand 18	
	CD163	CD163 molecule	
	ITGB5	Integrin subunit beta 5	

a Ma; Macrophage markers.

Multivariate normality was determined by the Shapiro–Wilk test and multicollinearity, homoscedasticity and extreme values were evaluated. We considered a *P *≤* *0.05 as statistically significant. R 3.1.1 was used for all statistical calculations.

## Results

### Group differences at baseline

The groups differed in several aspects at baseline (Table 1). Overweight, dysglycemic men had elevated levels of Hba1_c_, diastolic blood pressure, BMI, weight, fasting insulin and glucose levels, alanine transaminase (ALAT) and liver fat content, and lower GIR, compared to control participants (Table 1).

### Response to 12 w exercise

No weight change was observed in control men, whereas a reduction of 1.7 kg was observed in the dysglycemic men (Table 1). Both groups improved similarly in GIR and had reduced levels of liver fat content and plasma Fetuin‐A levels after the intervention (Table 1). The change in liver fat content was slightly different between the groups (Table 1). Plasma glucose levels increased slightly in controls and the change in plasma insulin levels differed between the groups (Table 1). The steady‐state insulin levels during the clamp were higher in the dysglycemic men after the intervention (Table 1). Plasma ALAT and aspartate transaminase (ASAT) levels were reduced in the dysglycemic men, also compared to the controls, after the 12 w intervention (Table 1). Plasma FFA levels were reduced in the control men after the intervention (Table 1), also compared to the dysglycemic men.

### Plasma levels of fetuin‐A and FFAs, and their interaction, related to changes in insulin sensitivity in response to 12 weeks exercise

**Figure 1:**
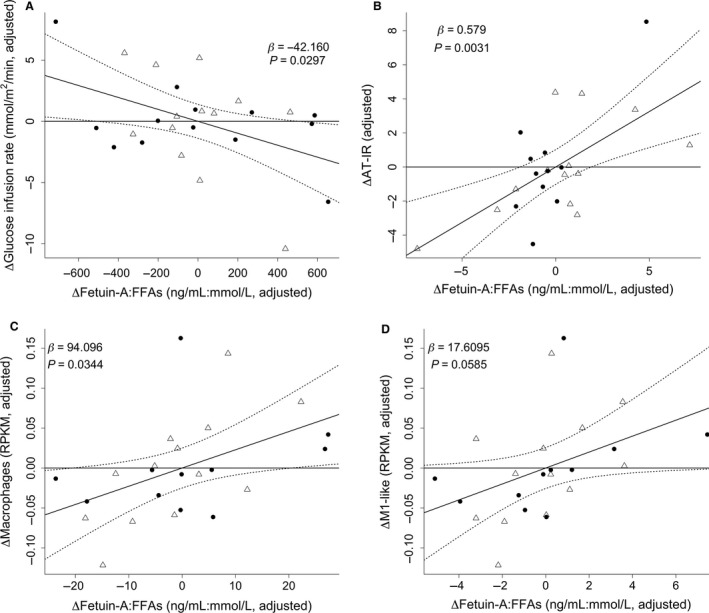
Interaction between changes in plasma concentration of fetuin‐A and FFAs predicted changes in insulin sensitivity, adipose tissue insulin resistance, and adipose tissue macrophage‐related gene expression in response to exercise. Leverage plots are presented and show the unique effect of the interaction term in multiple regression models. The constrained model without the interaction term is shown as a horizontal line; the unconstrained model with the interaction term is shown as a slanted line with associated 95% confidence intervals as stapled lines. “Adjusted” refers to covariates in the model, which included subjects, due to repeated measurements, and group allocation. Black circles refer to control men and open triangles refer to dysglycemic men. The interaction effect between changes in plasma concentration of fetuin‐A and FFAs negatively predicted changes in the glucose infusion rate (A) and positively predicted changes in adipose tissue insulin resistance (B), adipose tissue macrophage‐related gene expression (C), and tended to predict the M1‐like macrophage phenotype (D) in response to 12 weeks of exercise intervention. FFAs; free fatty acids, AT‐IR; adipose tissue insulin resistance, indicated by the product of fasting plasma FFA and insulin levels. The full regression models are presented in Table 2 and Table 6. The list of mRNA markers of macrophage‐related gene expressions in human adipose tissue is presented in Table 5. “:” is the Wilkinson‐Rogers symbolic description of factorial models for analysis of variance indicating an interaction.

Neither changes in plasma concentrations of fetuin‐A nor FFAs alone could predict changes in GIR in response to exercise (Table 2). However, the interaction between changes in plasma fetuin‐A and FFAs levels, representing the fetuin‐A‐fatty acid complex (Pal et al. 2012; Stefan and Haring 2013a) was a predictor of the change in GIR in response to exercise (Table 2). Similar observations were experienced using the product of fasting insulin and FFAs levels as a proxy for adipose tissue insulin resistance. No effects were observed for neither plasma fetuin‐A nor FFAs levels separately (data not shown), but a possible interaction effect was observed (Fig. 1B).

To test whether the observed relationships of FFA levels with GIR was specific for fetuin‐A, we investigated whether plasma concentrations of leptin and adiponectin would exhibit similar effects. There were no significant interaction effects between total or high molecular weight adiponectin and leptin with GIR or adipose tissue insulin resistance (data not shown).

### Relationships between changes in plasma levels of fetuin‐A and FFAs, and their interaction, with changes in TLR and insulin receptor signaling pathways

We examined whether plasma concentration of fetuin‐A and FFAs, and their interaction, predicted changes in gene expression related to TLR and insulin receptor signaling in adipose tissue (Bessman et al. 1986; Pal et al. 2012). As a comparison, we also performed the analysis on the skeletal muscle.

**Figure 2:**
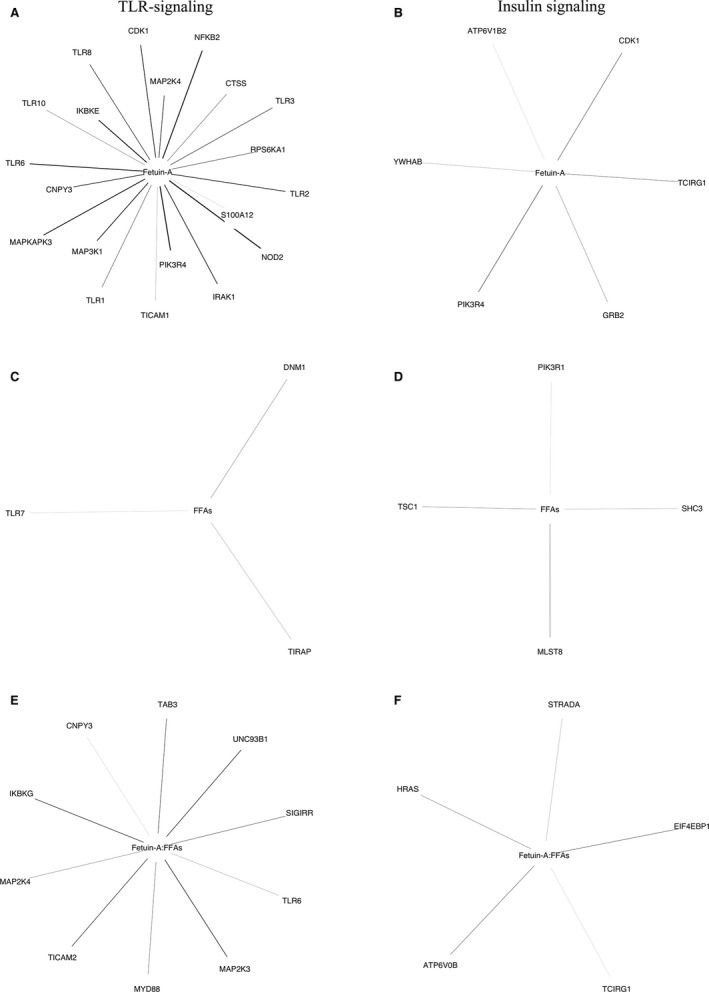
Genes involved in adipose tissue TLR‐ and insulin receptor signaling correlated with changes in plasma concentration of fetuin‐A and FFAs. (A) Genes involved in adipose tissue TLR‐signaling correlated with plasma fetuin‐A levels. (B) Genes involved in adipose tissue insulin receptor signaling correlated with plasma fetuin‐A levels. (C) Genes involved in adipose tissue TLR‐signaling correlated with plasma FFAs levels. (D) Genes involved in adipose tissue insulin receptor signaling correlated with plasma FFAs levels. (E) Genes involved in adipose tissue TLR‐signaling correlated with the interaction between plasma fetuin‐A and FFAs levels. (F) Genes involved in adipose tissue insulin receptor signaling correlated with the interaction between plasma fetuin‐A and FFAs levels. The relationship between gene expression and plasma concentration of fetuin‐A and FFAs was modeled using linear regression. The genes predicted by the model were overlapped with KEGG, Reactome, and Biocarta pathways related to TLR‐ and insulin receptor signaling using a hypergeometric distribution, to test if the overlap was larger than expected by chance. The statistics and more details regarding the pathway analyses are presented in the legend to Table 3 and the method section. Thinner lines indicate the strongest significance. “:” is the Wilkinson‐Rogers symbolic description of factorial models for analyses of variance indicating an interaction.

In the adipose tissue, plasma fetuin‐A levels alone, as well as and the interaction between plasma fetuin‐A and FFAs levels, predicted changes in TLR‐signaling on the mRNA level (Table 3). The genes within the TLR and insulin receptor signaling‐related gene sets, which correlated with plasma FFA and fetuin‐A levels, and their interaction, are presented in Figure 2.

**Figure 3:**
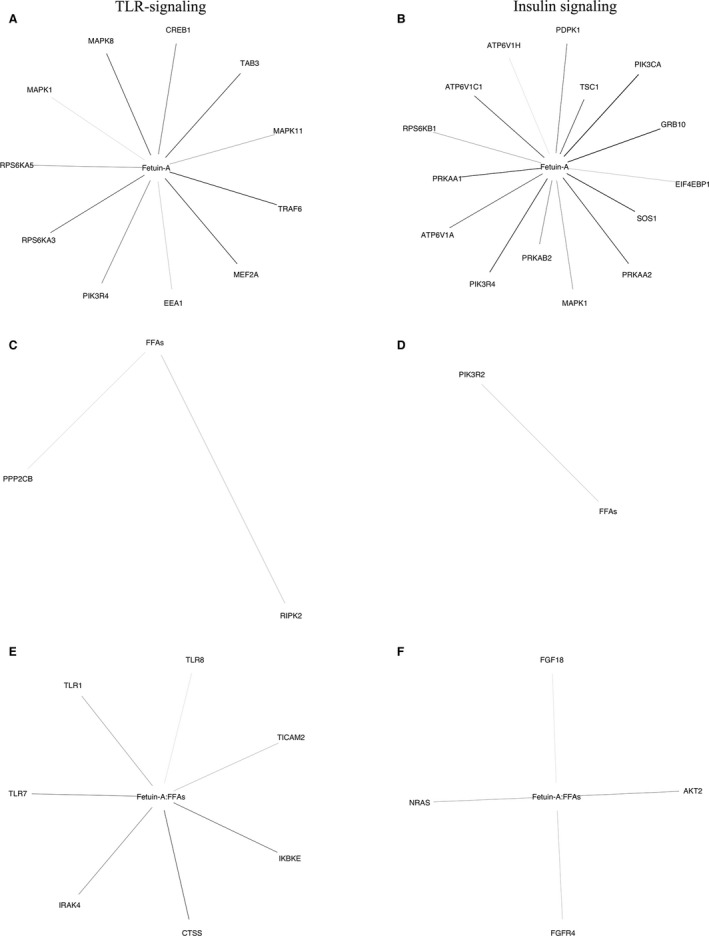
Genes involved in skeletal muscle TLR‐ and insulin receptor signaling related to changes in plasma concentration of fetuin‐A and FFAs. (A) Genes involved in skeletal muscle TLR‐signaling correlated with plasma fetuin‐A levels. (B) Genes involved in skeletal muscle insulin receptor signaling correlated with plasma fetuin‐A levels. (C) Genes involved in skeletal muscle TLR‐signaling correlated with plasma FFAs levels. (D) Genes involved in skeletal muscle insulin receptor signaling correlated with plasma FFAs levels. (E) Genes involved in skeletal muscle TLR‐signaling correlated with the interaction between plasma concentration of fetuin‐A and FFAs. (F) Genes involved in skeletal muscle insulin receptor signaling correlated with the interaction between plasma concentration of fetuin‐A and FFAs. The relationship between gene expression and plasma fetuin‐A and FFAs levels was modeled using linear regression. The genes significantly predicted by the model were overlapped with KEGG, Reactome, and Biocarta pathways related to TLR‐ and insulin receptor signaling using a hypergeometric distribution, to test if the overlap was larger than expected by chance. The statistics and more details regarding the pathway analyses are presented in the legend to Table 4. Thinner lines indicate the strongest significance. “:” is the Wilkinson‐Rogers symbolic description of factorial models for analysis of variance indicating an interaction.

Plasma concentration of fetuin‐A levels tended to predict TLR‐related gene expression in the skeletal muscle, and predicted insulin receptor signaling‐related gene expression (Table 4). The genes overlapping these pathways are presented in Figure 3. We observed no interaction effects between plasma concentration of fetuin‐A and FFAs on TLR and insulin receptor signaling in the skeletal muscle.

### Adipose tissue macrophages might be related to plasma concentrations of fetuin‐A and FFAs and their interaction

Adipose tissue macrophages are important regulators of metabolism and inflammation, and proinflammatory “M1‐like” macrophages are known to induce insulin resistance (Hill et al. 2014). Thus, we tested if the change in markers of adipose tissue macrophages (Table 5) in response to exercise could be predicted by plasma concentrations of fetuin‐A and FFAs levels and their interaction. Plasma concentration of fetuin‐A alone, but not FFA levels, might predict adipose tissue macrophage‐related gene expression and the M1‐like phenotype (Table 6 and Fig. 2). Moreover, interaction between plasma concentration of fetuin‐A and FFAs may also predict adipose tissue macrophage‐related gene expression and the M1‐like phenotype (Table 6 and Fig. 2).

**Table 6:** Relationships between plasma concentration of fetuin‐A and FFAs, and their interaction, with adipose tissue macrophage‐related gene expression

	Macrophages	*P*	M1‐like	*P*	M2‐like	*P*
	*β *± SE^a^		*β *± SE		*β *± SE	
Fetuin‐A						
(Intercept)	3.493 ± 4.981	0.491	0.830 ± 1.029	0.429	−0.182 ± 1.914	0.925
Group^b^	−10.387 ± 5.824	0.089	−0.954 ± 1.203	0.437	−4.443 ± 2.238	0.060
Fetuin‐A	30.328 ± 17.585	0.099	8.159 ± 3.633	0.036	4.052 ± 6.756	0.555
FFAs						
(Intercept)	−1.265 ± 4.652	0.788	−0.546 ± 1.003	0.592	0.095 ± 1.626	0.954
Group	−8.844 ± 6.371	0.180	−0.453 ± 1.374	0.745	−5.052 ± 2.226	0.034
FFAs	1.463 ± 4.642	0.756	0.189 ± 1.001	0.852	2.131 ± 1.622	0.203
Fetuin‐A*FFAs						
(Intercept)	3.290 ± 4.960	0.515	0.741 ± 1.051	0.490	0.527 ± 1.935	0.788
Group	−9.170 ± 5.707	0.125	−0.678 ± 1.210	0.582	−4.816 ± 2.227	0.043
Fetuin‐A	52.133 ± 19.027	0.013	12.268 ± 4.032	0.007	9.890 ± 7.423	0.199
FFAs	8.139 ± 5.116	0.128	1.403 ± 1.084	0.211	4.115 ± 1.996	0.053
Fetuin‐A:FFAs	94.096 ± 41.277	0.034	17.609 ± 8.748	0.059	26.964 ± 16.103	0.110

a SE; standard error.

b Dysglycemic, overweight participants (of which two participants did not donate adipose tissue biopsies). The list of mRNA markers used to analyze macrophage‐related gene expression in human adipose tissue is presented in Table 5. Plasma concentration of fetuin‐A and FFAs were log‐transformed to approximate multivariate normality.

## Discussion

Previous studies have mainly focused on the roles of myokines (Pedersen et al. 2007; Pedersen and Febbraio 2012) and adipokines (Gorgens et al. 2015; Stanford et al. 2015) to explain the health benefits of exercise. In this study, we demonstrate that long‐term physical exercise reduced plasma concentrations of the hepatokine fetuin‐A, suggesting that some of the health effects of exercise may be mediated via altered production and secretion of a hepatokine. Although it has been demonstrated that exercise may reduce plasma fetuin‐A concentrations (Malin et al. 2014), and that interaction of fetuin‐A and FFAs can induce TLR4‐associated insulin resistance (Pal et al. 2012; Stefan and Haring 2013a), we seem to be the first to demonstrate that changes in fetuin‐A and FFAs together may predict some of the benefits seen on insulin sensitivity after long‐term exercise. Moreover, we demonstrate that changes in circulating fetuin‐A together with FFAs may predict adipose tissue insulin resistance, gene expression concerning TLR‐signaling and macrophage‐related gene expression.

In accordance with the study of Malin and colleagues (Malin et al. 2014), we demonstrated that long‐term physical exercise reduced plasma concentrations of fetuin‐A. Interestingly, circulating fetuin‐A has also been shown to correlate strongly and positively with markers of early atherosclerosis (Rittig et al. 2009; Dogru et al. 2013) and components of the metabolic syndrome (Ix et al. 2006), independent of adiposity. Furthermore, circulating fetuin‐A levels correlated negatively with measures of insulin sensitivity (Mori et al. 2006; Ishibashi et al. 2010; Kantartzis et al. 2010; Xu et al. 2011; Kaess et al. 2012), and high circulating plasma fetuin‐A levels can be a strong predictor of T2DM incidence (Ix et al. 2008, 2012; Stefan et al. 2008; Sun et al. 2013). High plasma concentrations of fetuin‐A and FFAs have also been observed in insulin‐resistant individuals, and lower circulating fetuin‐A levels were observed after treatment with statins (Stefan and Haring 2013a). Thus, reducing the amount of circulating fetuin‐A by physical exercise might be important to prevent or treat T2DM and CVD.

Our data are compatible the idea that interaction between fetuin‐A and FFAs may represent the link between lipid metabolism and insulin resistance in humans in vivo. This is coherent with data indicating that interaction between fetuin‐A and FFAs was a predictor of insulin sensitivity (Stefan and Haring 2013a), and with studies on mice and different cell lines (Pal et al. 2012; Stefan and Haring 2013a). Fetuin‐A might also induce insulin resistance by itself, as well as together with FFAs, at least according to studies on overfeeding in humans (Samocha‐Bonet et al. 2014). This is also indicated by our gene expression results, revealing a link between fetuin‐A separately on skeletal muscle insulin receptor signaling and adipose tissue TLR receptor signaling. We hypothesize that less fetuin‐A is available after exercise, which might reduce the level of TLR4‐dependent insulin resistance in adipose tissue through lower plasma concentration of fetuin‐A and less interaction with FFAs. This might be reflected in increased insulin sensitivity following exercise. However, we cannot exclude that exercise alters plasma FFAs composition. Fetuin‐A binds several types of FFAs with different affinity, the strongest affinity being for saturated fatty acids such as palmitic acid (Pal et al. 2012; Stefan and Haring 2013a). Thus, alterations in plasma FFAs composition after exercise might thus influence the ability of fetuin‐A for inducing TLR4‐signaling.

The interaction between fetuin‐A and FFAs explained some of the variability in improving GIR in our study. GIR is mainly a measurement of skeletal muscle insulin sensitivity, although it is also markedly influenced by hepatic, and to a lesser degree, adipose tissue insulin sensitivity (DeFronzo et al. 1979). Measurements of endogenous glucose production, e.g., with glucose tracers would have strengthened our study, although hyperinsulinemia during clamp suppresses endogenous (hepatic) glucose production (DeFronzo et al. 1979; Reaven et al. 1983). However, we are unable to evaluate the degree of suppression as we did not measure hepatic insulin sensitivity directly, and 40 mU/m^2^ min^−1^ might not have been sufficient for insulin‐resistant participants. Some studies suggest that indices, such as the QUICKI (Katz et al. 2000) and MEC (Tajiri et al. 2011), which incorporates glucose and insulin levels during the clamp test in the formulas, might represent better measures of insulin sensitivity compared to GIR. However, GIR is the actual amount of glucose disappearing from blood without other variables in the calculations, thereby reducing sources of variation.

Another weakness in our study is the lack of measurement of FFAs levels during the clamp tests, which may provide an indication of adipose tissue insulin sensitivity. Instead, we calculated the product of fasting plasma concentrations of FFAs and insulin as a proxy. Taken together with the gene expression results, our study suggests that the interaction between fetuin‐A and FFAs relates to adipose tissue TLR‐signaling and adipose tissue insulin resistance. When looking for interaction between FFAs and other known cytokines on insulin sensitivity, such as adiponectin and leptin, we detected no such relationships. Moreover, we observed no interaction effect of fetuin‐A and FFAs on skeletal muscle TLR‐signaling. These results suggest a specific effect of fetuin‐A together with FFAs on insulin sensitivity related to the adipose tissue.

Adipose tissue macrophages may be important regulators of inflammation and metabolism (Hill et al. 2014). Our data and calculations based on results from previous studies (Capel et al. 2009; Ahlin et al. 2013; Hill et al. 2014), may suggest a relationship between plasma fetuin‐A and FFAs and adipose tissue macrophages. Fetuin‐A may promote inflammatory cytokine expression in monocytes and adipocytes, and may repress production of the insulin‐sensitizing adipokine adiponectin (Hennige et al. 2008; Dasgupta et al. 2010). Future studies should extend analyses of the relationship between adipose tissue macrophages and fetuin‐A and FFAs.

Some data indicate that increased plasma FFAs levels increase hepatic fetuin‐A expression in the liver by increasing NF‐*κ*B activity (Dasgupta et al. 2010), whereas other data suggest that high plasma glucose levels increase hepatic fetuin‐A expression in the liver via activation of the ERK‐1–ERK‐2 signaling pathway (Takata et al. 2009). However, the mechanisms behind the link between physical exercise and circulating fetuin‐A levels remain unknown. A weakness of our study is that we do not have liver biopsies and hence we were unable to assess the expression of the fetuin‐A gene directly.

In conclusion, we show that long‐term exercise may increase insulin sensitivity by lowering plasma concentration levels of the hepatokine fetuin‐A, probably by interfering with adipose tissue. Future studies should focus on identifying novel exercise‐regulated hepatokines with effect on energy metabolism.

## Conflict of Interest

The authors declare that there is no conflict of interest that could be perceived as prejudicing the impartiality of the reported research. The MyoGlu study is a registered clinical trial in US National Library of Medicine Clinical Trials registry (NCT01803568).

## References

[phy213183-bib-0001] Auberger, P. , L. Falquerho , J. O. Contreres , G. Pages , G. Le Cam , B. Rossi , et al. 1989 Characterization of a natural inhibitor of the insulin receptor tyrosine kinase: cDNA cloning, purification, and anti‐mitogenic activity. Cell 58:631–640. 2766355 10.1016/0092-8674(89)90098-6

[phy213183-bib-0002] Bessman, S. P. , C. Mohan , and I. Zaidise . 1986 Intracellular site of insulin action: mitochondrial Krebs cycle. Proc. Natl Acad. Sci. USA 83:5067–5070. 3523482 10.1073/pnas.83.14.5067 PMC323891

[phy213183-bib-0003] Capel, F. , E. Klimcakova , N. Viguerie , B. Roussel , M. Vitkova , M. Kovacikova , et al. 2009 Macrophages and adipocytes in human obesity: adipose tissue gene expression and insulin sensitivity during calorie restriction and weight stabilization. Diabetes 58:1558–1567. 19401422 10.2337/db09-0033 PMC2699855

[phy213183-bib-0004] Dasgupta, S. , S. Bhattacharya , A. Biswas , S. S. Majumdar , S. Mukhopadhyay , S. Ray , et al. 2010 NF‐kappaB mediates lipid‐induced fetuin‐A expression in hepatocytes that impairs adipocyte function effecting insulin resistance. Biochem. J. 429:451–462. 20482516 10.1042/BJ20100330

[phy213183-bib-0005] DeFronzo, R. A. , J. D. Tobin , and R. Andres . 1979 Glucose clamp technique: a method for quantifying insulin secretion and resistance. Am. J. Physiol. 237:E214–E223. 382871 10.1152/ajpendo.1979.237.3.E214

[phy213183-bib-0006] Dogru, T. , H. Genc , S. Tapan , F. Aslan , C. N. Ercin , F. Ors , et al. 2013 Plasma fetuin‐A is associated with endothelial dysfunction and subclinical atherosclerosis in subjects with nonalcoholic fatty liver disease. Clin. Endocrinol. 78:712–717. 10.1111/j.1365-2265.2012.04460.x 22676641

[phy213183-bib-0007] Finucane, M. M. , G. A. Stevens , M. J. Cowan , G. Danaei , J. K. Lin , C. J. Paciorek , et al. 2011 National, regional, and global trends in body‐mass index since 1980: systematic analysis of health examination surveys and epidemiological studies with 960 country‐years and 9.1 million participants. Lancet (London, England) 377: 557–567. 21295846 10.1016/S0140-6736(10)62037-5 PMC4472365

[phy213183-bib-0008] Fu, S. , S. M. Watkins , and G. S. Hotamisligil . 2012 The role of endoplasmic reticulum in hepatic lipid homeostasis and stress signaling. Cell Metab. 15:623–634. 22560215 10.1016/j.cmet.2012.03.007

[phy213183-bib-0009] Gorgens, S. W. , K. Eckardt , J. Jensen , C. A. Drevon , and J. Eckel . 2015 Exercise and regulation of adipokine and myokine production. Prog. Mol. Biol. Transl. Sci. 135:313–336. 26477920 10.1016/bs.pmbts.2015.07.002

[phy213183-bib-0010] Heinrichsdorff, J. , and J. M. Olefsky . 2012 Fetuin‐A: the missing link in lipid‐induced inflammation. Nat. Med. 18:1182–1183. 22869185 10.1038/nm.2869

[phy213183-bib-0011] Hennige, A. M. , H. Staiger , C. Wicke , F. Machicao , A. Fritsche , H. U. Haring , et al. 2008 Fetuin‐A induces cytokine expression and suppresses adiponectin production. PLoS ONE 3:e1765. 18335040 10.1371/journal.pone.0001765 PMC2258416

[phy213183-bib-0012] Hill, A. A. , W. Reid Bolus , and A. H. Hasty . 2014 A decade of progress in adipose tissue macrophage biology. Immunol. Rev. 262:134–152. 25319332 10.1111/imr.12216 PMC4203421

[phy213183-bib-0013] Ishibashi, A. , Y. Ikeda , T. Ohguro , Y. Kumon , S. Yamanaka , H. Takata , et al. 2010 Serum fetuin‐A is an independent marker of insulin resistance in Japanese men. J. Atheroscler. Thromb. 17:925–933. 20543523 10.5551/jat.3830

[phy213183-bib-0014] Ix, J. H. , M. G. Shlipak , V. M. Brandenburg , S. Ali , M. Ketteler , and M. A. Whooley . 2006 Association between human fetuin‐A and the metabolic syndrome: data from the Heart and Soul Study. Circulation 113:1760–1767. 16567568 10.1161/CIRCULATIONAHA.105.588723 PMC2776669

[phy213183-bib-0015] Ix, J. H. , C. L. Wassel , A. M. Kanaya , E. Vittinghoff , K. C. Johnson , A. Koster , et al. 2008 Fetuin‐A and incident diabetes mellitus in older persons. JAMA 300:182–188. 18612115 10.1001/jama.300.2.182 PMC2779582

[phy213183-bib-0016] Ix, J. H. , M. L. Biggs , K. J. Mukamal , J. R. Kizer , S. J. Zieman , D. S. Siscovick , et al. 2012 Association of fetuin‐a with incident diabetes mellitus in community‐living older adults: the cardiovascular health study. Circulation 125:2316–2322. 22511752 10.1161/CIRCULATIONAHA.111.072751 PMC3390925

[phy213183-bib-0017] Jung, T. W. , H. J. Yoo , and K. M. Choi . 2016 Implication of hepatokines in metabolic disorders and cardiovascular diseases. BBA Clin. 5:108–113. 27051596 10.1016/j.bbacli.2016.03.002 PMC4816030

[phy213183-bib-0018] Kaess, B. M. , D. M. Enserro , D. D. McManus , V. Xanthakis , M. H. Chen , L. M. Sullivan , et al. 2012 Cardiometabolic correlates and heritability of fetuin‐A, retinol‐binding protein 4, and fatty‐acid binding protein 4 in the Framingham Heart Study. J. Clin. Endocrinol. Metabol. 97:E1943–E1947. 10.1210/jc.2012-1458 PMC3674297 22855337

[phy213183-bib-0019] Kantartzis, K. , J. Machann , F. Schick , A. Fritsche , H. U. Haring , and N. Stefan . 2010 The impact of liver fat vs visceral fat in determining categories of prediabetes. Diabetologia 53:882–889. 20099057 10.1007/s00125-010-1663-6

[phy213183-bib-0020] Katz, A. , S. S. Nambi , K. Mather , A. D. Baron , D. A. Follmann , G. Sullivan , et al. 2000 Quantitative insulin sensitivity check index: a simple, accurate method for assessing insulin sensitivity in humans. J. Clin. Endocrinol. Metab. 85:2402–2410. 10902785 10.1210/jcem.85.7.6661

[phy213183-bib-0021] Langleite, T. M. , J. Jensen , F. Norheim , H. L. Gulseth , D. S. Tangen , K. J. Kolnes , et al. 2016 Insulin sensitivity, body composition and adipose depots following 12 w combined endurance and strength training in dysglycemic and normoglycemic sedentary men. Arch. Physiol. Biochem. 1–13. 10.1080/13813455.2016.1202985 27477619

[phy213183-bib-0022] Lee, S. , F. Norheim , T. M. Langleite , H. J. Noreng , T. H. Storas , L. A. Afman , et al. 2016 Effect of energy restriction and physical exercise intervention on phenotypic flexibility as examined by transcriptomics analyses of mRNA from adipose tissue and whole body magnetic resonance imaging. Physiol. Rep. 4. 10.14814/phy2.13019 PMC5112497 27821717

[phy213183-bib-0023] Li, T. Y. , J. S. Rana , J. E. Manson , W. C. Willett , M. J. Stampfer , G. A. Colditz , et al. 2006 Obesity as compared with physical activity in predicting risk of coronary heart disease in women. Circulation 113:499–506. 16449729 10.1161/CIRCULATIONAHA.105.574087 PMC3210835

[phy213183-bib-0024] Malin, S. K. , J. P. del Rincon , H. Huang , and J. P. Kirwan . 2014 Exercise‐induced lowering of fetuin‐A may increase hepatic insulin sensitivity. Med. Sci. Sports Exerc. 46:2085–2090. 24637346 10.1249/MSS.0000000000000338 PMC4640446

[phy213183-bib-0025] Mori, K. , M. Emoto , H. Yokoyama , T. Araki , M. Teramura , H. Koyama , et al. 2006 Association of serum fetuin‐A with insulin resistance in type 2 diabetic and nondiabetic subjects. Diabetes Care 29:468. 16443916 10.2337/diacare.29.02.06.dc05-1484

[phy213183-bib-0026] Nolan, C. J. , P. Damm , and M. Prentki . 2011 Type 2 diabetes across generations: from pathophysiology to prevention and management. Lancet (London, England) 378: 169–181. 21705072 10.1016/S0140-6736(11)60614-4

[phy213183-bib-0027] Olefsky, J. M. , and C. K. Glass . 2010 Macrophages, inflammation, and insulin resistance. Annu. Rev. Physiol. 72:219–246. 20148674 10.1146/annurev-physiol-021909-135846

[phy213183-bib-0028] Ouchi, N. , J. L. Parker , J. J. Lugus , and K. Walsh . 2011 Adipokines in inflammation and metabolic disease. Nat. Rev. Immunol. 11:85–97. 21252989 10.1038/nri2921 PMC3518031

[phy213183-bib-0029] Pal, D. , S. Dasgupta , R. Kundu , S. Maitra , G. Das , S. Mukhopadhyay , et al. 2012 Fetuin‐A acts as an endogenous ligand of TLR4 to promote lipid‐induced insulin resistance. Nat. Med. 18:1279–1285. 22842477 10.1038/nm.2851

[phy213183-bib-0030] Pedersen, B. K. , and M. A. Febbraio . 2012 Muscles, exercise and obesity: skeletal muscle as a secretory organ. Nature Rev. Endocrinol. 8:457–465. 22473333 10.1038/nrendo.2012.49

[phy213183-bib-0031] Pedersen, B. K. , T. C. Akerstrom , A. R. Nielsen , and C. P. Fischer . 2007 Role of myokines in exercise and metabolism. J. Appl. Physiol. (Bethesda, Md: 1985) 103: 1093–1098. 17347387 10.1152/japplphysiol.00080.2007

[phy213183-bib-0032] Reaven, G. M. , J. Moore , and M. Greenfield . 1983 Quantification of insulin secretion and in vivo insulin action in nonobese and moderately obese individuals with normal glucose tolerance. Diabetes 32:600–604. 6345239 10.2337/diab.32.7.600

[phy213183-bib-0033] Rittig, K. , C. Thamer , A. Haupt , J. Machann , A. Peter , B. Balletshofer , et al. 2009 High plasma fetuin‐A is associated with increased carotid intima‐media thickness in a middle‐aged population. Atherosclerosis 207:341–342. 19615685 10.1016/j.atherosclerosis.2009.05.018

[phy213183-bib-0034] Ahlin, S. , K. Sjoholm P. Jacobson , J. C. Andersson‐Assarsson , A. Walley , J. Tordjman , C. Poitou , et al. 2013 Macrophage gene expression in adipose tissue is associated with insulin sensitivity and serum lipid levels independent of obesity. Obesity (Silver Spring, Md) 21: E571–E576. 23512687 10.1002/oby.20443 PMC3763968

[phy213183-bib-0035] Saltiel, A. R. , and C. R. Kahn . 2001 Insulin signalling and the regulation of glucose and lipid metabolism. Nature 414:799–806. 11742412 10.1038/414799a

[phy213183-bib-0036] Samocha‐Bonet, D. , C. S. Tam , L. V. Campbell , and L. K. Heilbronn . 2014 Raised circulating fetuin‐a after 28‐day overfeeding in healthy humans. Diabetes Care 37:e15–e16. 24356603 10.2337/dc13-1728

[phy213183-bib-0037] Stanford, K. I. , R. J. Middelbeek , and L. J. Goodyear . 2015 Exercise effects on white adipose tissue: beiging and metabolic adaptations. Diabetes 64:2361–2368. 26050668 10.2337/db15-0227 PMC4477356

[phy213183-bib-0038] Stefan, N. , and H. U. Haring . 2013a Circulating fetuin‐A and free fatty acids interact to predict insulin resistance in humans. Nat. Med. 19:394–395. 23558619 10.1038/nm.3116

[phy213183-bib-0039] Stefan, N. , and H. U. Haring . 2013b The role of hepatokines in metabolism. Nat. Rev. Endocrinol. 9:144–152. 23337953 10.1038/nrendo.2012.258

[phy213183-bib-0040] Stefan, N. , A. Fritsche , C. Weikert , H. Boeing , H. G. Joost , H. U. Haring , et al. 2008 Plasma fetuin‐A levels and the risk of type 2 diabetes. Diabetes 57:2762–2767. 18633113 10.2337/db08-0538 PMC2551687

[phy213183-bib-0041] Sun, Q. , M. C. Cornelis , J. E. Manson , and F. B. Hu . 2013 Plasma levels of fetuin‐A and hepatic enzymes and risk of type 2 diabetes in women in the U.S. Diabetes 62:49–55. 22923470 10.2337/db12-0372 PMC3526056

[phy213183-bib-0042] Tajiri, Y. , S. Sato , and K. Yamada . 2011 Metabolic clearance rate is a more robust and physiological parameter for insulin sensitivity than glucose infusion rate in the isoglycemic glucose clamp technique. Diabetes Technol. Ther. 13:1057–1061. 21714676 10.1089/dia.2011.0042

[phy213183-bib-0043] Takata, H. , Y. Ikeda , T. Suehiro , A. Ishibashi , M. Inoue , Y. Kumon , et al. 2009 High glucose induces transactivation of the alpha2‐HS glycoprotein gene through the ERK1/2 signaling pathway. J. Atheroscler. Thromb. 16:448–456. 19672022 10.5551/jat.no950

[phy213183-bib-0044] Turer, A. T. , and P. E. Scherer . 2012 Adiponectin: mechanistic insights and clinical implications. Diabetologia 55:2319–2326. 22688349 10.1007/s00125-012-2598-x

[phy213183-bib-0045] Xu, Y. , M. Xu , Y. Bi , A. Song , Y. Huang , Y. Liu , et al. 2011 Serum fetuin‐A is correlated with metabolic syndrome in middle‐aged and elderly Chinese. Atherosclerosis 216:180–186. 21310413 10.1016/j.atherosclerosis.2011.01.020

[phy213183-bib-0046] Zimmet, P. , K. G. Alberti , and J. Shaw . 2001 Global and societal implications of the diabetes epidemic. Nature 414:782–787. 11742409 10.1038/414782a

